# The Risky Health Behaviours of Male Adolescents in the Southern Italian Region: Implications for Sexual and Reproductive Disease

**DOI:** 10.3390/jcm8091414

**Published:** 2019-09-08

**Authors:** Anna Perri, Danilo Lofaro, Giulia Izzo, Benedetta Aquino, Massimo Bitonti, Giuseppe Ciambrone, Sandro La Vignera, Carlotta Pozza, Daniele Gianfrilli, Antonio Aversa

**Affiliations:** 1 Kidney and Transplantation Research Center, Department of Nephrology, Dialysis and Transplantation, Annunziata Hospital, 87100 Cosenza, Italy; 2 Department of Experimental and Clinical Medicine, Magna Græcia University, 88100 Catanzaro, Italy; 3 Procrea Private Practice Association, 88100 Catanzaro, Italy; 4 AOU Materdomini, 88100 Catanzaro, Italy; 5 Department of Clinical and Experimental Medicine, University of Catania, 95124 Catania, Italy; 6 Department of Experimental Medicine, Sapienza University of Rome, 00185 Rome, Italy

**Keywords:** adolescent, lifestyle, fertility, transitional age

## Abstract

Recent epidemiological studies suggest an increase of sexual and reproductive chronic diseases caused by problematic behaviours acquired during peri-pubertal age. The aims of our study were: (i) to investigate awareness of sexual transmitted infections (STIs) among adolescents; (ii) to describe the close relationship between possibly incorrect lifestyles during adolescence and reproductive and sexual disturbances during adulthood. The “Amico-Andrologo” survey is a permanent nationwide surveillance program supported by the Italian Ministry of Health. We administered a validated structured interview to investigate the lifestyle of adolescents and their knowledge of STIs. We selected a cohort of 360 male high-school students aged ≥18 years old. In this cohort, 150 (41.5%) were smokers while 59 (19.7%) smoked more than 10 cigarettes/day; 25 (9.3%) declared a consumption ≥6 drinks/weekend; and 65 (19.7%) were habitual cannabis consumers (at least twice/week). Among the sample of students selected, the main sources of sexual disease information were the internet and friends. The perceived level of knowledge on STIs was the same between students that used contraceptive methods and students that did not. The present results demonstrate that adolescents in Calabria do not receive appropriate information about risky health behaviours. Therefore, there is a necessity for specific educational programs to increase awareness of dangerous behaviours during the transitional age that is relevant for a safe sexual and reproductive adult life.

## 1. Introduction

“Risky behaviours” are habits that may impact future well-being and are typical for adolescents [1,2]. The transition from childhood to adulthood is determined by a series of psycho-physical and hormonal changes. These changes are fundamental for the complete maturation of teenagers and for acceptance and respect by their peers [3]. It is well known that adolescence is crucial for the correct development and maturation of the genitourinary tract. Epidemiological data have demonstrated an increase in chronic sexual and reproductive diseases caused by risky behaviours developed during adolescence [4,5]. Alcohol and marijuana are the most common substances abused by adolescents [6]. On the one hand, ethanol interferes not only with the production of gonadal steroids by blocking GnRH cascade, but also increases the oxidative stress inducing damage of Leydig and Sertoli cell functions. This may lead to lower semen volume and sperm motility and morphological alterations [7]. On the other hand, cannabis, although it raises sexual desire, may hamper erectile function [8]. In fact, cannabis interferes with endothelial nitric oxide release, leading to vascular alterations in the absence of other risk factors [9]. In addition, the use of these substances often facilitates adolescents to have unprotected sexual intercourse with different partners, thus increasing their exposure to unwanted pregnancies and to Sexually Transmitted Infections (STIs) [10]. Studies on the sexual activity of Italian adolescents has shown that the age of one’s first sexual intercourse is low (about 15.6 years old), and that 19.5% of new STI cases involve the youngest sexually active group (15–24 years old) [11,12]. STIs may contribute to male infertility, with Chlamydia trachomatis, Neisseria gonorrhoeae, and Herpes Papilloma Virus (HPV) infection being the most frequent pathogens in up to 15% of cases [13,14]. Even a heavy cigarette smoking habit may affect penile vasculature, reduce testosterone levels, and increase oxidative stress; therefore, cigarette use can influence semen volume and quality, leading to infertility [15].

In order to reduce the development of risky behaviours during adolescence, a series of health prevention and intervention campaigns have been conducted in the US. A first level approach was the Condom distribution program intended to prevent STIs [16]. A second, but no less important approach, was to encourage teens’ communication with their extended family. Such relationships should be scheduled in teen health programs, which primarily focus on parents [17]. Although this latter aspect is considered a novel way to counteract risky behaviours among adolescents, it is not easily applied in specific social contexts.

Unfortunately, according to our knowledge, there are only a few adequate prevention programs aimed at changing sexual and reproductive attitudes among Italian teenagers, particularly in Southern Italian areas. The current study is a part of a nationwide andrological health surveillance program that was carried out on a sample of male adolescents attending their last year of High School in Calabria. The instrument of our survey was the administration of a questionnaire (an adaptation of the Centers for Disease Control and Prevention (CDC) Youth Risk Behaviours Surveillance (YRBS)) to estimate the prevalence of health risk behaviours.

## 2. Experimental Section

### 2.1. Subjects and Methods

The “Amico-Andrologo” Survey is a permanent national project conducted by the Italian Society of Andrology and Sexual Medicine (SIAMS) on young male adolescents attending the last year of high school, which was approved by the local Institutional Board Review, Regional School Authorities, and the Ministry of Health (protocol 19251/P—“Prevenzione in Andrologia” signed 28 April 2008). All male subjects were invited to complete an anonymous, self-administered, written questionnaire.

Between September 2016 and May 2017, we enrolled 360 male students (≥18 years old) attending high schools in the district of Cosenza, Catanzaro and Reggio di Calabria, Calabria area, Italy. The Survey investigated the adolescents’ lifestyles (smoking habits, alcohol and drug consumption, and sexual behaviours and experiences) and explored the students’ knowledge about STIs and major sources of information about these issues.

### 2.2. Statistical Analysis

All data are presented as the mean ± SD or count (%), as appropriate. A multiple comparison was performed using the Kruskal–Wallis test and Conover–Iman test for pairwise comparisons. The questionnaire data were collected and statistically elaborated by the R software (Version 3.5.3, The R Foundation for Statistical Computing).

## 3. Results

### Health Risk Behaviors

The demographic characteristics of the participants are reported in Table 1. Since the adolescents interviewed were attending their last high-school year, they were all aged 18 or 19 (61.3% and 39.7%, respectively). Students enrolled were normal-weighted (BMI = 22.6 ± 3.1). According to their family situation, we discovered that 87 students (24.2%) had separated or divorced parents, and 14 (3.9%) had lost one or both of their parents. However, we found no association between demographic characteristics and substance abuse or problematic sexual habits.

**Figure 1:**
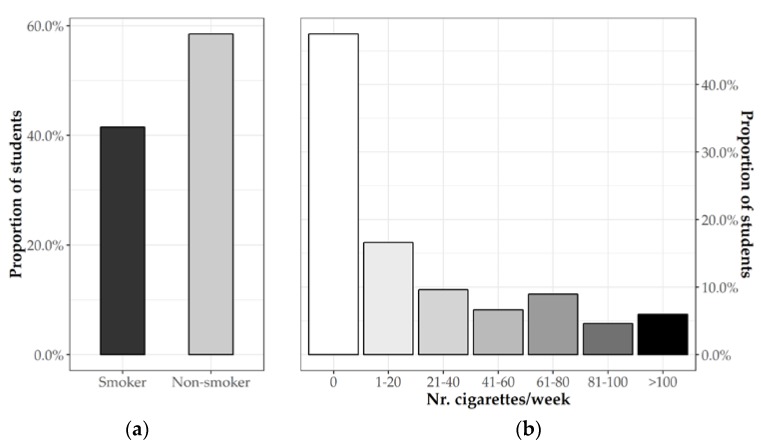
(**a**) Percent of smokers and non-smokers students and (**b**) number of cigarettes/day.

The analysis of smoking habits revealed that, overall, 150 students (41.5%) reported smoking (Figure 1a). In more detail, 59 students (19.7%) reported smoking approximately 10 or more cigarettes/day (Figure 1b), and, surprisingly, 124 students (35.2%) smoked their first cigarette before 15 years of age (Table 2).

**Figure 2:**
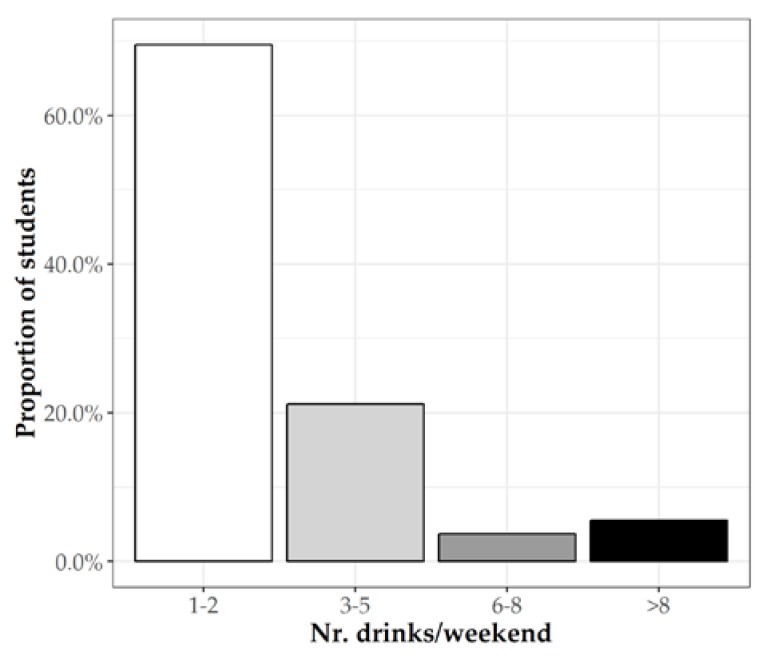
Number of alcoholic drinks/weekend.

According to alcohol consumption, only 25 students (9.3%) declared a consumption of ≥6 drinks per weekend (Figure 2).

**Figure 3:**
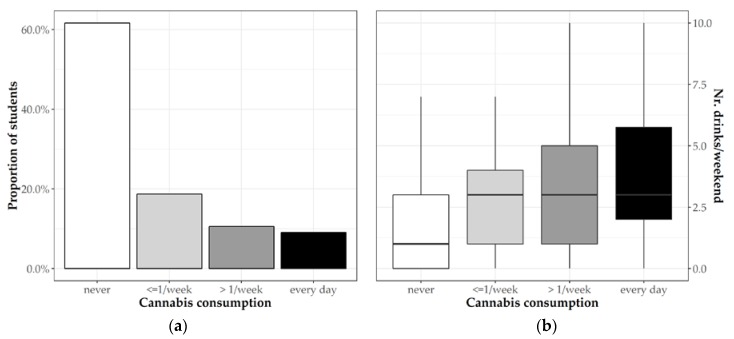
(**a**) Frequency of cannabis consumption among students and (**b**) the association between alcohol and cannabis consumption.

For the consumption of illicit drugs, 65 students (19.7%) declared to be habitual cannabis smokers (at least twice a week; Figure 3a). Moreover, we found a linear association between cannabis and alcohol consumption (*p* < 0.001; Figure 3b).

**Figure 4:**
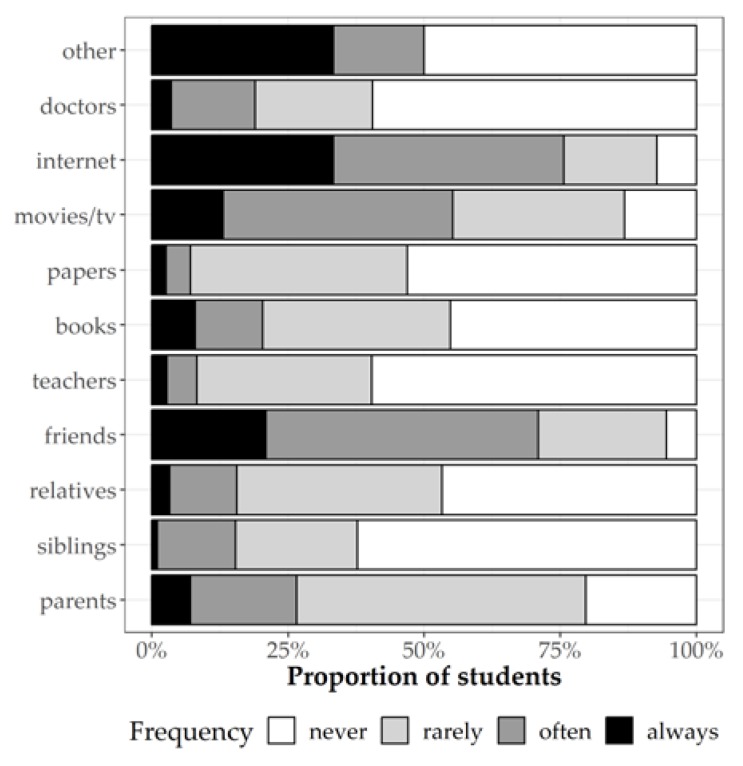
Percentage of the students’ reported source for information on sexuality.

Data on sexual education showed that the internet and peers were the main sources of information about sexuality, contraception, and STIs. On the other hand, only a few students stated that they had received sexual education from teachers, doctors, or relatives (Figure 4).

**Figure 5:**
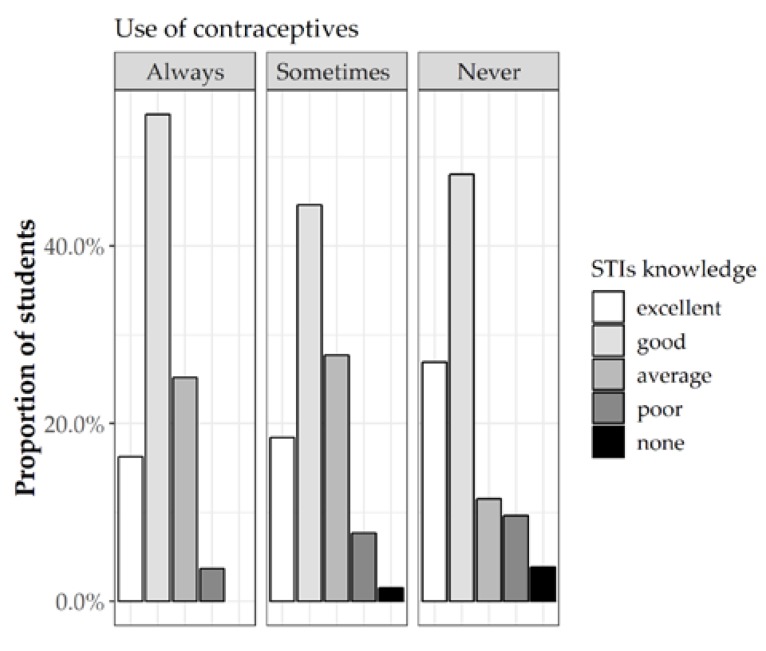
Perceived level of knowledge on STIs between students according to contraceptive use frequency.

Interestingly, 242 (53.5%) of the 360 sexually active students examined admitted they use condoms; 65 students (25.6%) used contraceptive sometimes, while the remaining 53 (20.9%) declared they had never used them. Most of the students reported sufficient information about STIs: 61 students (17.7%) showed excellent knowledge, while 169 (49.0%) reported good knowledge. Surprisingly, these percentages were higher among those not using contraceptives at all (Figure 5).

## 4. Discussion

Our study is the first to show data on male adolescents’ attitudes and risky behaviours in a Southern Italian region (Calabria). The results demonstrate that their consumption of cannabis and alcohol is lower compared to that of their peers living in Central or Northern Italian regions. Moreover, we found out that most of these adolescents receive information on sexuality and STIs mainly from the internet and friends and that a good knowledge of STIs was, surprisingly, associated with a habit to not use contraceptive methods.

It is well known that adolescence is associated with high levels of alcohol binge drinking because it encourages the initiation of sexual activity by lowering anxiety and inhibitions [18]. Unfortunately, chronic alcohol consumption is also associated with the appearance of sexual dysfunction, such as the loss of libido, premature ejaculation, and erectile dysfunction [19]. Adolescents also admit to the regular use of cannabis and other illicit drugs, risky behaviours that expose them to potential future sexual disturbances and diseases [20]. In addition, consumption of these substances is often continued throughout their adult lives, with further negative consequences on sexuality and fertility [9].

In contrast with our results, Gianfrilli et al. have recently reported that in several Northern and Central Italian regions, just one over half (51%) of the subjects examined admitted to being regular cigarette smokers. Even the percentages related to occasional alcohol consumption (80%) and heavy drinking habits (30%) were much higher when compared to the results of our study [21]. One point in common with Gianfrilli’s study is that adolescents try illegal drugs, especially marijuana/hashish, at an early age. Cigarette smokers are also equally represented in all Italian regions. The consumption of alcohol (per weekend = 9.3%) and cannabis (least once-a-week = 21%) were lower in Southern regions than in Northern ones. Interestingly, we observed that the use of alcohol and cannabis among adolescents in Southern regions is often combined. This finding had already been reported by other authors in a survey conducted on young people ages 13 to 19 [22].

Luckily, recent studies have demonstrated that, since the early 2000s, the consumption of tobacco, alcohol, and cannabis among adolescents is decreasing both in Europe and North America [23,24]. In fact, the International Health Behaviour in School-aged Children (HBSC) study showed that the percentage of 15 year old smokers in Europe and North America decreased from 24% in 2002 to 12% in 2014. Weekly alcohol consumption decreased from 29% to 13%, and, finally, the habit of cannabis smoking among 15 year old boys decreased from 22% to 15% [25,26]. These changes may be due to a sharp drop in face-to-face peer contacts that yielded an increased use of electronic media communications [27]. The results of the studies conducted in Northern, Central, and Southern Italian regions are in contrast with these findings and, therefore, suggest that Italian adolescents need more information and awareness about the dangers of risky behaviours. Thus, it is advisable to promote a series of robust campaigns in Italian regions aimed at changing the attitudes of adolescents regarding the excessive use of tobacco/marijuana.

Gianfrilli et al. showed that 60.3% of adolescents engage in regular sexual activity and, among them, 41% have unprotected sex [21]. Our results highlight a discrepancy between the belief that adolescents have good knowledge of the risks related to unprotected sex and their habit not to use condoms, even with different partners. Indeed, although about 70% of the respondents declared to know about STIs, we observed that 25.6% of students examined used contraceptive “sometimes” and that the 20.9% of them used contraceptives “never”. These findings are in line with reported data in the literature. The survey conducted by Drago et al. on 2867 Italian adolescents, which aimed to investigate knowledge about sexuality, revealed that 22% of the interviewees knew that condoms are the only contraceptive means for preventing STIs, and just 0.5% have recognized sexual diseases on the list provided [11]. Similar results have been reported by Bergamini et al, whose study pointed out the need to improve teenagers’ awareness of risky behaviours for the prevention of STIs. In their opinion, school should play a crucial role in the reinforcement of sexual educational programs [28]. The survey of Wand et al., carried out on a sample of 16–29 year old subjects, demonstrated that the majority of high risk sexual behaviours and STI diagnoses were associated with the consumption of illicit drugs and alcohol. In conclusion, the diffusion of STIs could be prevented by mitigating high risky sexual behaviours among teenagers [29].

As we have already stated before, another risky behaviour among adolescents is represented by their attitude to have unprotected sexual intercourse. This dangerous habit predisposes male adolescents to the development of male accessory gland infections (MAGI), including prostatitis, vesiculitis, and/or epididymitis. Several kinds of microorganisms are involved in the etiology of MAGI, such as *Escherichia coli*, *Neisseria gonorrhoeae*, *Chlamydia trachomatis*, *Ureaplasma urealyticum*, *Mycoplasma hominis*, and other mycoplasmas, *Candida albicans*, *Trichomonas vaginalis*, and HPV [30]. We believe that a good information campaign is necessary both for families and adolescents, to contrast problematic habits in sexual intercourse. It is also advisable that HPV vaccination in 11 year old boys become mandatory in order to prevent the spread of STIs and other diseases not directly related to HPV. This new practice would likely contribute to a sharp reduction in the chronic, often asymptomatic, damage responsible for apparently idiopathic sperm abnormalities. Asymptomatic or pauci-symptomatic forms can lead to chronic inflammatory processes, responsible for the development of hypertrophic-congestive and fibro-sclerotic ultrasound-evidenced variants. These forms differently impact sperm quality, sometimes irreversibly [19]. They also may contribute to sexual dysfunction, such premature ejaculation [31].

In general, although the attitudes and sexual behaviours of the population have radically changed, adolescents usually approach sexuality unprepared and uninformed, especially when they live in underdeveloped socio-economic and cultural contexts. In addition, as we have already discussed, a high percentage of teenagers learn about these issues on the internet and compare what they know with their friends [32]. Unfortunately, the internet is just a container of general (and sometimes incorrect) information, ranging from pornography to (un)controlled medical indications that cannot provide the web-surfer with adequate interpretative tools. Several studies have shown that interpersonal relationships are the primary elements in the decision-making of adolescents in risky sexual behaviours and that peers are more important than adults in defining norms and attitudes [10,33].

We are aware that the limitations of the present study are related to the lack of data on testicular volume, sperm parameters and sexual function. We also recognize that a multicenter prospective study should be carried out to assess the impact of risky health behaviours on sexuality and fertility per year of exposure, starting from adolescence. However, we have to point out that this study, which is unique and relevant because of the novelty of its results, represents a different approach compared to previous epidemiological data coming from prevention campaigns in Calabrian areas.

## 5. Conclusions

Data from the present survey suggest that in Calabria, risky health behaviours are wide spread among male teenagers. Adolescents still do not acquire appropriate education about sexual and reproductive health behaviours because of their unreliable sources of information—peers or the internet, instead of institutional figures. Another negative attitude that affects adolescents’ sexual and reproductive lives is represented by their high alcohol and cannabis consumption during the convivial weekend meetings.

Therefore, it is necessary to promote focused educational programs, starting post-puberty, in order to make families and teenagers aware of the impact of MAGI on male fertility and sexuality.

We also recommend sensitizing young boys to the use of condoms, to mitigate the high spread of HPV and other STIs. Regarding this aspect, HPV vaccination in 11 year old boys could represent a fundamental element for the primary prevention of STIs, and even of other diseases not directly related to HPV.

In conclusion, we suggest involving families and schoolteachers in future information campaigns addressed to teenagers. A correct institutional information campaign is, in fact, the only way to produce a safe sexual and reproductive life in adulthood.

## Figures and Tables

**Table 1:** Clinical Features of enrolled students.

	Students (*n =* 360)
Age	
18	217 (61.3)
19	143 (39.7)
Weight (kg)	71.2 ± 10.6
High (cm)	177 ± 7.2
BMI (kg/m^2^)	22.6 ± 3.1
Parental Divorce/Separation	87 (24.2)
Fatherless and/or motherless	14 (3.9)

**Table 2:** Percentage of students divided by the different age of their first cigarette.

Age at First Cigarettes (%)	Students
never	114 (32.4)
≤8 (years)	5 (1.4)
9–10 (years)	12 (3.4)
11–12 (years)	36 (10.2)
13–14 (years)	71 (20.2)
15–16 (years)	85 (24.1)
≥17 (years)	29 (8.2)
